# Three-dimensional label-free morphology of CD8 + T cells as a sepsis biomarker

**DOI:** 10.1038/s41377-023-01309-w

**Published:** 2023-11-07

**Authors:** MinDong Sung, Jong Hyun Kim, Hyun-Seok Min, Sooyoung Jang, JaeSeong Hong, Bo Kyu Choi, JuHye Shin, Kyung Soo Chung, Yu Rang Park

**Affiliations:** 1 Division of Pulmonary and Critical Care Medicine, Department of Internal Medicine, Yonsei University College of Medicine, Seoul, Republic of Korea; 2 Department of Biomedical Systems Informatics, Yonsei University College of Medicine, Seoul, Republic of Korea; 3 Tomocube, Inc, 155 Sinseong-ro, Shinsung-dong, Yuseong-gu, Daejeon, Republic of Korea

**Keywords:** High-field lasers, Imaging and sensing

## Abstract

Sepsis is a dysregulated immune response to infection that leads to organ dysfunction and is associated with a high incidence and mortality rate. The lack of reliable biomarkers for diagnosing and prognosis of sepsis is a major challenge in its management. We aimed to investigate the potential of three-dimensional label-free CD8 + T cell morphology as a biomarker for sepsis. This study included three-time points in the sepsis recovery cohort (*N* = 8) and healthy controls (*N* = 20). Morphological features and spatial distribution within cells were compared among the patients’ statuses. We developed a deep learning model to predict the diagnosis and prognosis of sepsis using the internal cell morphology. Correlation between the morphological features and clinical indices were analysed. Cell morphological features and spatial distribution differed significantly between patients with sepsis and healthy controls and between the survival and non-survival groups. The model for predicting the diagnosis and prognosis of sepsis showed an area under the receiver operating characteristic curve of nearly 100% with only a few cells, and a strong correlation between the morphological features and clinical indices was observed. Our study highlights the potential of three-dimensional label-free CD8 + T cell morphology as a promising biomarker for sepsis. This approach is rapid, requires a minimum amount of blood samples, and has the potential to provide valuable information for the early diagnosis and prognosis of sepsis.

## Abstract (web summary)

This study discover the potential of using 3D label-free CD8 + T cell morphology as a reliable biomarker for the diagnosis and prognosis of sepsis, highlighting its rapid analysis and minimal blood sample requirements.

## Graphical abstract

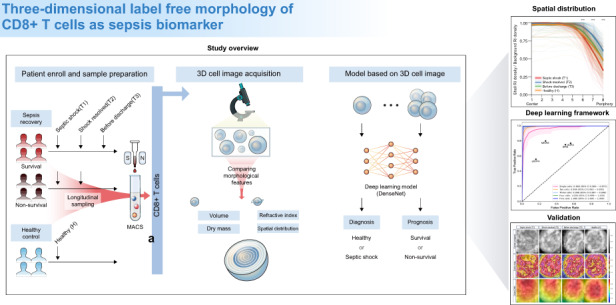

## Introduction

Sepsis is a “life-threatening organ dysfunction caused by the dysregulated host response to infection”^1^. Sepsis has high mortality rates^2,3^, and sepsis-specific treatment is lacking. One reason is that the immune response to sepsis is complex and varies among patients^4^. Therefore, making an early diagnosis and taking quick action is crucial because even a delay of 1 h in interventions can result in increased mortality^5^.

Numerous specific sepsis biomarkers have been recognised to enhance diagnostic accuracy, facilitate early recognition of organ dysfunction, assist in risk stratification, and monitor an individual’s immune response^6,7^. These biomarkers should deliver prompt results, allow for recurrent measurements, and accurately reflect the real-time status of the patient, ensuring that any changes in the condition are immediately mirrored in the biomarker reading, thus enabling timely interventions. However, commonly used biomarkers such as C-Reactive Protein (CRP)^8,9^ and Procalcitonin (PCT)^10,11^ have limitations, such as delayed responses. This delay arises from the time required for transcription in response to cytokines secreted by immune cells, followed by translation and protein synthesis^12–14^. Cytokines such as Interleukin-6 (IL-6) have been suggested as potential biomarkers to decrease this time lag. However, IL-6 presents its own challenges due to the lack of standardization and high susceptibility to other influences, which can make interpretation difficult^15^. Efforts have been made to further reduce this time lag, such as using RNA levels as biomarkers^16,17^ and leveraging single-cell sequencing to identify sepsis-specific immunologic signatures^18^. However, the extensive time required for RNA sequencing and analysis limits its immediate use as a biomarker. Newly developed biomarkers, including histidine-rich glycoprotein^19,20^, calprotectin^21^, and HMGB-11^22^, and newer technologies, such as microfluidic^23^, which can speed up these measurements still exhibit an inherent time lag, a common shortcoming of secretory molecules.

Despite the potential of the current biomarkers, their inherent limitations necessitate exploring novel approaches to identify sepsis biomarkers. One promising avenue is the study of immune cell morphology. These morphological changes in immune cells occur swiftly upon the onset of inflammation, closely mirror the patient’s immune status, and provide an immediate snapshot of the cellular responses to inflammation^24–27^. Advancements in microscopic techniques have enabled rapid and repeated measurements. However, most studies rely primarily on cell lines or healthy human cells for experimentation, which may not fully capture the complex dynamics of human immune responses. Therefore, more comprehensive investigations are needed to address these constraints and accurately depict the intricate dynamics of immune cells following sepsis.

We investigated the potential of immune cellular structures as a biomarker in sepsis based on the two technologies within a human sepsis sample: holotomography imaging and deep learning. Traditional staining and fixation methods may alter the native state of cells, inhibit dynamic cellular studies, and are time-consuming^28–30^. Holotomography imaging circumvents these drawbacks by providing label-free three-dimensional (3D) images of live cells and enabling an unaltered, real-time study of cells. This advanced technique measures a quantifiable physical parameter, the refractive index (RI), which correlates with cellular biochemical and biophysical properties^31,32^. Simultaneously, deep learning advancements have simplified the feature engineering process, and improved overall analysis accuracy^33,34^.

In this study, we employed label-free 3D imaging to analyze changes in immune cell structures, from healthy states to sepsis diagnosis, and throughout the sepsis recovery period. We identified noticeable alterations in the morphological features such as the volume and dry mass of CD8 + T cells, by comparing the data between healthy controls and patients with sepsis at different recovery stages. Additionally, we studied the spatial distribution within cells, revealing intriguing disparities in the cellular structures related to sepsis. To handle the substantial data derived from these 3D cellular structures, we utilized a deep learning model that demonstrated good performance in sepsis diagnosis and prognosis prediction. We also explored the correlation between cell morphology and clinical outcomes of sepsis, furthering our understanding of this relationship. Finally, we validated our deep learning model using interpretable algorithms to maintain transparency, suggesting that our methodology can be integrated effectively into clinical settings.

## Results

**Fig. 1:**
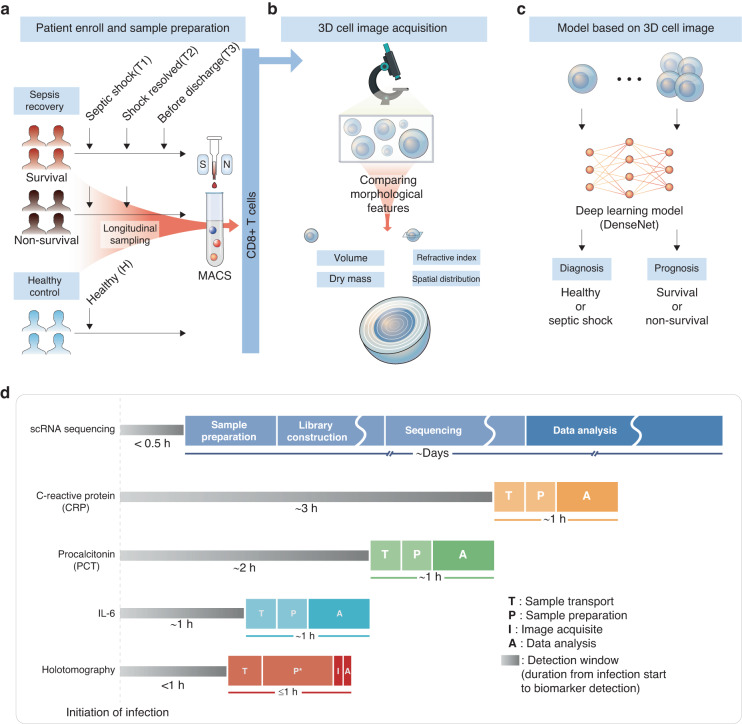
Schematic overview of the study workflow. **a** This study included sepsis recovery and healthy control. The sepsis recovery cohorts were classified into survival and non-survival groups. Blood was sampled three times in the sepsis recovery cohort and once in the healthy control cohort. CD8 + T cells were extracted from the blood using a Magnetic Cell Separator (MACS). **b** 3D cell images were acquired using holotomography. The morphological features of the 3D cell and the 2D sectioned images were obtained and compared. The spatial distribution within the cell was compared with that of the shell structure. **c** Deep learning models for predicting the diagnosis and the prognosis of sepsis were developed and validated based on internal cell structure. PBMCs, peripheral blood mononuclear cells; ICU, intensive care unit. **d** This schematic view compares the timeline of currently used biomarkers, including C-reactive protein (CRP), procalcitonin (PCT), and IL-6, with our proposed method (holotomography). Also, comparing the scRNA sequencing method. The diagram, highlighting the steps of sample transport (T), sample preparation (P), image acquisition (I), and data analysis (A), emphasizes the duration from the initiation of infection to the detection of biomarkers (Detection window). It also underscores the time taken for each step, showcasing the efficiency of our approach. scRNA, single cell RNA

Our study workflow, as illustrated in Fig. 1, involved enrolling sepsis patients with sepsis and collecting their clinical data along with blood samples at three crucial time points: upon diagnosis of septic shock (T1), following resolution of the septic shock (T2), and immediately before discharge (T3). Patients’ survival outcomes were recorded. Blood samples were obtained from healthy participants to serve as a control group. We isolated CD8 + T cells from these blood samples using magnetic-activated cell sorting (Fig. 1a), followed by obtaining label-free 3D imaging using a holotomography microscope (Fig. 1b). This process enabled us to extract biophysical features such as cell volume, refractive index (RI), and dry mass and to study the spatial distribution of internal cell components based on their RI values. Using these data, we constructed two distinct deep-learning models. The first model was designed to diagnose sepsis by differentiating healthy controls and patients with sepsis. The second model aimed to predict prognosis by distinguishing between survivors and non-survivors of sepsis patients. Both models are shown in Fig. 1c.

The primary advantage of our workflow is its exceptional ability to diagnose sepsis and predict prognosis using just a single cell or a few cells, thereby providing a crucial tool for sepsis management. Given that the time for image capture and analysis amounts to approximately less than 10 min, the overall process would take about an hour. For CRP, PCT, and IL-6 provide results in an hour, but the time from initiation of infection to biomarker detection takes about 1–3 h. RNA sequencing can detect the response immediately, but the process from preparation to data analysis can take several days (Fig. 1d).

### Participant characteristics

Our study enrolled 28 patients, including eight patients with sepsis and 20 healthy controls. The median age of the participants was 54 years, and 17 patients (61%) were female. In the sepsis cohort, three (11%), three (11%), and two (7.1%) patients were diagnosed with biliary, pneumonia, and urinary septic shock, respectively. The Sequential Organ Failure Assessment (SOFA) scores at time points 1, 2, and 3 were 8, 2, and 0, respectively. A total of 6198 3D images were obtained for analysis (Table 1).

**Table 1:** Demographic characteristics, and the total number of cell images acquired in sepsis recovery and healthy control cohorts

	Overall (*N* = 28)	Healthy (*N* = 20)	Sepsis (*N* = 8)
Age, year	54 (35, 72)	39 (34, 56)	78 (74, 88)
Male, *n* (%)	11 (39%)	7 (35%)	4 (55%)
Diagnosis			
Biliary septic shock, *n* (%)	3 (11%)	-	3 (38%)
Pneumonia septic shock, *n* (%)	3 (11%)	-	3 (38%)
Urinary septic shock, *n* (%)	2 (7.1%)	-	2 (25%)
Mechanical ventilator, *n* (%)	1 (12%)	-	1 (12%)
Renal replacement therapy, *n* (%)	1 (12%)	-	1 (12%)
1st time point SOFA^*^	8.00 (7.75, 9.75)	-	8.00 (7.75, 9.75)
2nd time point SOFA^*^	2.00 (1.00, 5.25)	-	2.00 (1.00, 5.25)
3rd time point SOFA^*^	0.00 (0.00, 1.50)	-	0.00 (0.00, 1.50)
ER^†^ length of stay, days	1.28 (0.91, 1.50)	-	1.28 (0.91, 1.50)
Total length of stay (days)	15 (10, 23)	-	15 (10, 23)
Number of total cells	6198	3335	2863

^*^SOFA Sequential Organ Failure Assessment, ^†^*ER* emergency room

### CD8 + T cell morphological changes through sepsis recovery

**Fig. 2:**
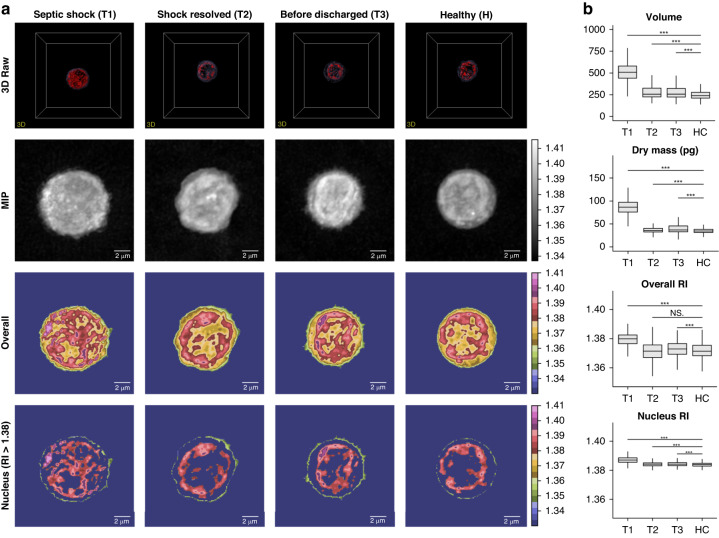
Overview of morphology and morphological features of cells in septic shock recovery and healthy control **a** 3D raw image, mid-point optical sectioned image, overall component coloured, and nucleus component coloured cell morphology in each status were drawn. **b** Boxplot and significance were plotted for each feature: volume, dry mass, overall, and nucleus RI distribution. 3D three-dimensional, MIP Maximal Intensity Projection, RI refractive index

In our study, we examined the morphological differences in CD8 + T cells during sepsis recovery compared with those in healthy controls. Figure 2a illustrates these differences in 3D cells (first row), optical sections of these images (second row), and colour maps of the overall and nuclear components (third and fourth rows).

We assessed the morphological features of cells at different time points during septic shock recovery and in healthy controls. Both cell volume (F-value 1043, *P* value < 2e-16) and dry mass (F-value 2174, *P* value < 2e-16) exhibited significant differences between the sepsis recovery time points and the healthy controls. Furthermore, the mean values of the overall (F-value 336.2, *P* value < 2e-16) and nuclear component RI (F-value 836.3, *P* value < 2e-16) demonstrated significant differences between sepsis recovery time points and healthy controls (Fig. 2b). We conclude that the significant morphological differences observed in CD8 + T cells during sepsis recovery and in healthy controls highlight their potential as biomarkers for the diagnosis and monitoring of sepsis progression.

### Spatial distribution within cells

**Fig. 3:**
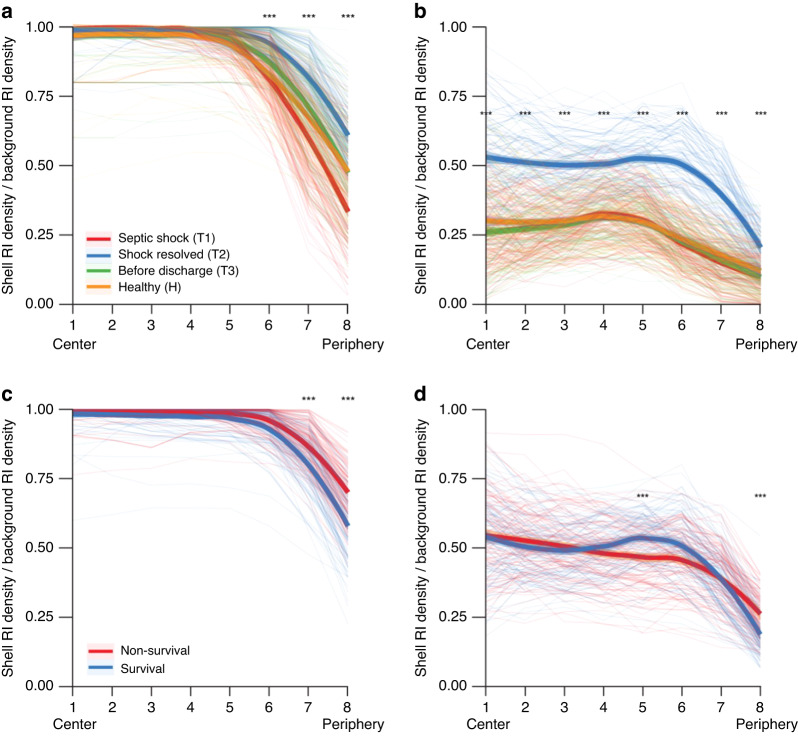
Spatial distribution within cells at each time point in longitudinal sepsis recovery and healthy control. Spatial distribution within cells in survival and non-survival groups at the first time point of sepsis recovery (T1) with the density of shell structure. The 2D sectioned images were divided into eight elliptically shaped regions, with the region closest to the centre referred to as Shell 1 and the region furthest from the centre referred to as Shell 8. The shell density is defined as the ratio of pixels with a value above the target RI to the total number of pixels in that shell. **a** The shell density of the overall RI at each time point of sepsis recovery and the healthy cohort. **b** Shell density of the nuclear component RI distribution at each time point of sepsis recovery and the healthy cohort. **c** Shell density of overall RI in the survival and non-survival groups at T1. **d** The shell density of the nuclear component RI in survivors and non-survivors at T1. ***p-value was <0.001. RI relative intensity, T1 time point 1, T2 time point 2, T3 time point 3 for sepsis recovery

In septic shock (T1), the spatial distribution within cells showed that the decreasing points existed more peripherally at T1 than that in the other statuses. In Shell 6, 7, and 8, there was a difference in shell density among the statuses (*P* value = 1.80e-15, 1.02e-35, and 3.70e-24, respectively) (Fig. 3a). In the nuclear component, the shell component density was higher in all shells, and the decrease in density in Shell 6 was slightly more peripheral than that in the other groups (Shell 4). These results indicated that the cell and nuclear sizes were larger and the components were denser at the septic shock time point (T1) compared to other status (Fig. 3b).

Moreover, comparing survival and non-survival at the septic shock time point (T1), the overall shell density showed differences in Shells 7 and 8 (*P* value = 0.0003 and 9.50e-10, respectively), resulting in the decreasing points existing more peripherally in the non-survival group than in the survival group. This indicated that the number of cells was higher in the non-survival group than in the survival group (Fig. 3c). The nuclear component showed no consistent pattern between the two groups, with significant differences in Shells 5 and 8 (*P* value = 0.0006 and 1.15e-15, respectively) (Fig. 3d).

### Deep learning models for predicting the diagnosis and prognosis of sepsis

**Fig. 4:**
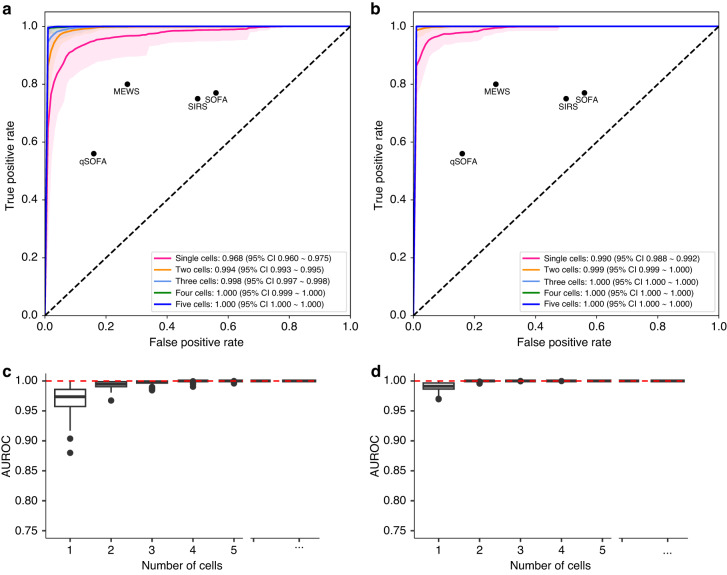
The receiver operating characteristic (ROC) curve of the proposed method with one–five cells in predicting diagnosis and prognosis models. The ROC curve of the model predicts (**a**) diagnosis, which classifies between septic shock status and healthy controls, and (**b**) prognosis, which classifies between the survival and the non-survival groups. The AUROC and associated 95% confidence intervals are included. The black dots depict the precision and recall rates of each clinical index used to predict the diagnosis and prognosis of sepsis. Boxplot of AUROCs for predicting (**c**) diagnosis, and (**d**) prognosis when cells were randomly sampled. AUROC, Area Under the Receiver Operating Characteristic curve; SIRS, Systemic Inflammatory Response Syndrome ; SOFA, Sequential Organ Failure Assessment; qSOFA, Quick Sequential Organ Failure Assessment; MEWS, Modified Early Warning Score; CI, confidence interval

The spatial distribution within cells was significantly different among patients with sepsis. Based on this knowledge, we used a deep learning model to extract morphological features efficiently. In predicting the diagnosis, nearly 100% of the area under the receiver operating characteristic (AUROC) curve was shown in the model that differentiated cells between septic shock (T1) in sepsis recovery cohorts and healthy controls (H) with a few cells. Our model performed significantly better than established clinical indices such as SIRS, qSOFA, SOFA, and MEWS with AUROC values of 0.70, 0.77, 0.78, and 0.50, respectively, for diagnosis^35^ (Fig. 4a). Furthermore, in the model that predicted prognosis with the cells in T1, the performance showed nearly 100% AUROC with a few cells. The predicted prognostic performances of the known indices, including SIRS, qSOFA, SOFA, and MEWS was 0.63, 0.52, 0.68, and 0.64, respectively (Fig. 4b). Even the model with a single cell showed better performance than the other clinical indices in predicting diagnosis and prognosis. When we examined the AUROC using random sampling to account for possible selection bias, we observed that the range of the AUROCs decreased as the number of cells increased, indicating that the more cells were selected, the less selection bias and variance was identified (Fig. 4c, d). These results suggest that we uncovered the potential of deep learning models using CD8 + T cells morphological features as a promising method to diagnose sepsis more accurately and predict its prognosis.

### Validation with clinical features and a visual explanation

**Fig. 5:**
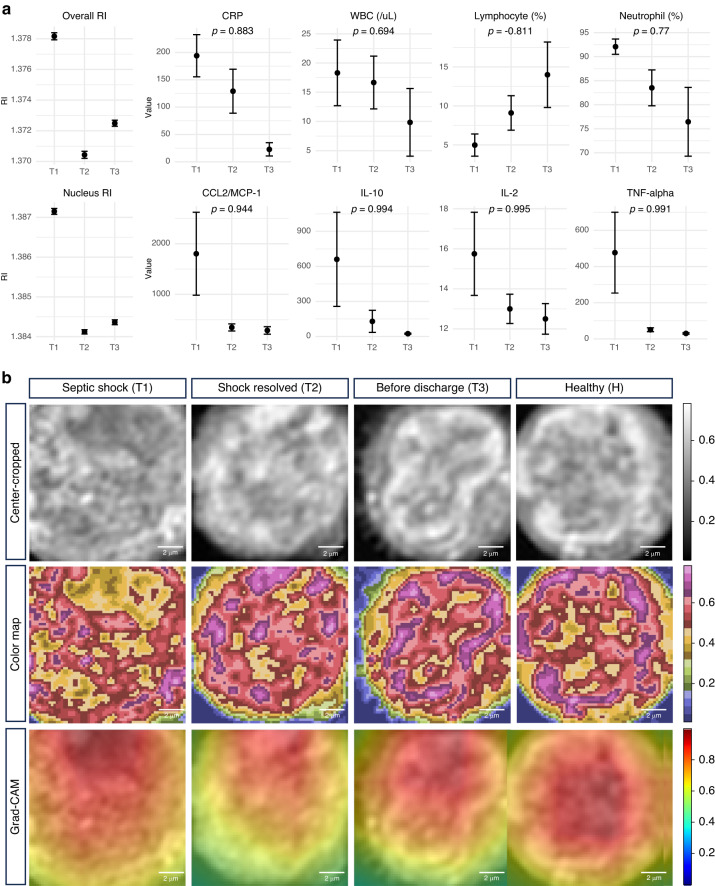
Validation of the morphological feature through correlation with clinical features and the visual explanation. **a** The scatter plot with an error bar of the mean RI, clinical features, including CRP, WBC count, lymphocytes (%), neutrophils (%), and cytokines, including CCL2/MCL-1, IL-10, IL-2, and TNF-alpha, at each time point of sepsis recovery. Additionally, the correlation coefficient (*ρ*) is described on each clinical feature and cytokine plot. **b** A cross-section of the sample images at each time point of sepsis recovery and healthy control is plotted. The colour-mapped images of the same cell image are plotted below. Additionally, the Grad-CAM images, superimposed on the original image, are plotted in the bottom row. RI, refractive index; CRP, C-reactive protein; WBC, white blood cell; IL, interleukin; TNF, Tumour necrosis factor; Grad-CAM, Gradient-weighted Class Activation Mapping

We observed that the morphological features and other clinical features, such as laboratory test results, including white blood cells (WBC), neutrophils, lymphocytes, CRP, and cytokine levels, were highly correlated. Laboratory tests related to inflammation in sepsis, including WBC, CRP, and neutrophils, decreased in sepsis recovery over time, except for lymphocytes. Additionally, the levels of the cytokines, including CCL2/MCP-1, IL-10, IL-2, and TNF-alpha, decreased. The morphological feature in each time point showed a similar pattern with the laboratory test and cytokines, which showed a high correlation (CRP 0.88, WBC (/µL) 0.68, neutrophil (%) 0.76, lymphocyte (%) 0.81, CCL2/MCP-1 0.944, IL-10 0.994, IL-2 0.995, and TNF-alpha 0.991) with a mean value of each time point lab values (Fig. 5a).

Moreover, we applied the Grad-CAM algorithm to understand which regions of the cell images were the most important for the prediction using the deep learning model. Figure 5b shows representative cell images with the accompanying saliency heat maps, highlighting the features that had the greatest influence on the model’s prediction. In the heat maps, the model prediction was based on features within the cells. The heat maps varied among the different time points, indicating that the model could recognise the changes within the cells. We discovered a strong correlation between cell morphology and clinical features, indicating the promising role of cellular biomarkers and deep learning models in advancing the diagnosis, prognosis, and understanding of the underlying biological processes of sepsis.

## Discussion

This study demonstrated that 3D label-free CD8 + T cell morphology could be a potential biomarker for sepsis. These morphological features of immune cells are potential biomarkers of sepsis because they are rapid, require the least number of samples, and can differentiate between different states of sepsis. We observed that the morphological features of CD8 + T cells differed between the time points of sepsis recovery and healthy control. Using spatial distribution analysis, the cells and nuclei at the first time point of sepsis recovery, which was septic shock, showed a larger volume and higher RI than those at other time points and in healthy controls. Moreover, the cell volume in the non-survival group was larger than in the survival group at the first time point of recovery from sepsis. Because the spatial distribution within a cell is an important feature, we developed a deep learning model based on the 3D cellular structures that can predict the diagnosis and prognosis of sepsis. The performance of models showed a nearly 100% AUROC for predicting events with a few cells. The diagnostic model achieved an AUROC of 99.7%, whereas the prognostic model archieved a performance of 99.9%. Furthermore, cell morphological features such as CRP, WBC count, lymphocytes, and cytokines were highly correlated with the clinical features used by physicians when making decisions in patients with sepsis. Therefore, 3D label-free CD8 + T cell structures are amenable to periodic and repeated testing of patient samples, as they require only a drop of blood for rapid measurement.

When compared to currently used biomarkers such as CRP, PCT, and IL-6, our methodology considerably reduces the time lag between the initiation of the infection and the response. Our method also overcomes the limitations of the sequencing approaches which currently lack the capacity for immediate detection of RNA expression or biomarkers upon infection initiation. Enhancements in our technique have allowed for speedy and frequent measurements, further reducing this time lag. Furthermore, we anticipate that the efficiency of our workflow could be considerably amplified with upcoming advancements in sample preparation techniques. As such, we see an extensive potential for the expansion of its practical applications in the future.

In sepsis diagnosis and prognosis prediction, conventional clinical indices such as MEWS, SIRS, qSOFA, and SOFA^35^ are widely used. However, these have several limitations. First, their prediction performance is often only reaches less than 80% of the AUROC performance^20^. Secondly, although patients may have the same clinical scores, sepsis symptoms, severity, and progression may vary. The use of rule-based scores may be insufficient to capture patient-specific statuses.

Several recent studies have been conducted to diagnostic and prognostic prediction models for sepsis. Goh et al.^36^ used both structured and unstructured data from electronic medical records (EMRs) to predict early sepsis. They observed that the performance of the model, measured by the AUROC metric, was high at 0.94 at the time of diagnosis; however, it decreased as the prediction time increased. Hu et al.^37^ developed a prognosis prediction model using machine learning with 57 clinical parameters. They predicted hospital mortality and showed an AUROC of 0.884. However, EMRs can have time delays in data entry and availability, which can affect the accuracy of predictions.

Our method, using a few CD8 + T cell morphologies, achieved an average AUROC performance of nearly 100% for both diagnosis and predicted prognosis of sepsis. This high performance underscores that cell morphology can serve as a highly accurate and efficient biomarker for sepsis.

In this study, the changes in the shape of the cells were clear as sepsis recovered, particularly when viewed centrally at the periphery of the cells. At time point 1 of sepsis recovery, the RI values were higher than those in the other states, and the nuclear compartments also increased. Furthermore, the RI values of the non-survival groups at time point 1 of sepsis recovery status were higher than those of the survivor group. This is thought to reflect changes in T-cell activation. A central DNA pattern was observed during T-cell activation, and nuclear size increased, which is likely related to T-cell differentiation^38^. In addition, lymphocytes from infected or vaccinated animals have a high RI due to increased protein concentrations resulting from the production of antibodies^39,40^.

Our study demonstrated a strong correlation between the morphological features, clinical features, and cytokines. Clinical features and cytokines showed a decreasing pattern throughout recovery from sepsis, except for an increasing pattern in lymphocytes. This suggests that cell morphology has the potential to predict clinical features and could be used to predict patient status. Moreover, using Grad-CAM, it is clear that the deep learning model focuses on the internal regions of the cells when making predictions.

Similar to all other studies, our study has some limitations. First, the intervals between the blood samples were not consistent. This could potentially affect the accuracy and comparability of the results. However, the recovery time for each patient varied, and the duration was based more on whether the patients recovered from shock rather than on a specific time interval. That is, recovery status was defined by considering the patient’s condition; resolved septic shock (T2) was defined as tapering out the vasopressor, and discharge (T3) as recovery from organ failure, rather than a consistent time interval, to accurately reflect the patient’s recovery. Second, the age differed between the sepsis recovery and healthy cohorts. As people get younger, they tend to have fewer comorbidities, making them a more accurate healthy control group. Additionally, strict criteria were used for the sepsis group to eliminate the differences caused by comorbidities, immunocompromised, or immunosuppressed status. This led to a significant age difference between the healthy control and sepsis groups; however, this was thought to help demonstrate the differences between the two groups.

In conclusion, 3D label-free CD8 + T cells can be used as biomarkers to predict the diagnosis and prognosis of patients with sepsis. Our findings demonstrate that CD8 + T cell morphology can reflect changes in sepsis status during recovery and help predict the prognosis of patients with sepsis. This efficient and accurate method has the potential to assist in the clinical management of patients with sepsis. Furthermore, sepsis is considered an important disease in which immune dynamics play a significant role. From this perspective, the use of immune cell morphology as a biomarker of sepsis could potentially be expanded to other immune-related diseases.

## Materials and methods

### Participant enrollment

This study included two cohorts: sepsis recovery cohort and a healthy control cohort. The sepsis recovery cohort was recruited from the emergency department of Severance Hospital between April and June 2022. They consisted of patients diagnosed with sepsis as defined by the Sepsis-3 consensus definitions^41^. Immunocompromised patients and those taking immunosuppressants were excluded (see Section 1 of the Supplementary Information). Clinical data were collected for each individual, and blood was sampled at three-time points: septic shock diagnosis (T1), septic shock resolution (T2), and before discharge (T3). The healthy cohort (H) comprised 20 healthy volunteers as the control group. The sampled blood was processed into peripheral blood mononuclear cells (PBMC), and the CD8 + T cells were extracted through magnetic-activated cell separation.

Informed consent was obtained from all participants. This study was approved by the Institutional Review Board of Severance Hospital, Yonsei University Health System, Seoul, Korea (IRB nos. 4-2021-1236 and 4-2022-0317).

### Plasma and peripheral blood mononuclear cell (PBMC) isolation

Peripheral whole blood was collected in EDTA tubes and processed fresh using Ficoll‒Paque Plus separation (GE Healthcare, Barrington, IL, USA, 17144002). The blood was first diluted with 5 mL of 2 mM EDTA‒PBS (Invitrogen, Carlsbad, CA, USA, 1555785-038) before 10–20 mL of diluted blood was carefully layered onto 15 mL of Ficoll in a 50 mL Falcon tube. The samples were centrifuged at 900 g for 30 min at room temperature (22 °C–24 °C). The plasma layer was carefully separated, and the PBMC layer was collected using a sterile Pasteur pipette. The PBMC layer was washed with three volumes of EDTA-PBS by centrifugation at 500 g for 5 min. The pellet was resuspended in EDTA-PBS and centrifuged at 400 g for 5 min. The PBMC pellet was collected, and the cell number and viability were assessed using Trypan blue and a Countess II Automated Cell Counter (Thermo Fisher Scientific, Waltham, MA, USA).

### Cell sorting

Cells were sorted using magnetic-activated cell sorting (Miltenyi Biotec, Bergisch Gadbach, Germany), a typical method for isolating the cells from a mixed population. The CD8 + T cells were negatively selected. The isolated cells were maintained at 4 °C to maintain cell viability. Then, 80 µL of isolation buffer and 20 μL microbeads were added, and the cells were incubated for 15 min at 4 °C. By setting the magnetic stand and column for sorting, we first equilibrated the column by washing it with 3 mL of isolation buffer. We withdrew the column from the magnetic rack to collect each lymphocyte and set up a conical tube. Next, 5 mL of isolation buffer was added, and the solution was pumped through the column to extract the final sorted cells from the collection tube.

### Cell image acquisition and processing

Three-dimensional (3D) cell images of CD8 + T cells were obtained using 3D holotomography (HT-2H; Tomocube Inc., Daejeon, Republic of Korea), which reconstructs a 3D RI image using multiple 2D quantitative phase images^42^. Cellular RI is an intrinsic optical parameter that determines how light travels through the cell-matrix, demonstrated through physical phenomena such as the scattering and absorption of light. RI is closely linked to the amount and distribution of cellular mass. We manually filtered out low-quality, low-resolution, noisy background images and images in which two or more cells were too closely adjoined.

For the deep learning process, we used a pre-processing method that considers the size of each 3D cell image. We first applied a predefined threshold to each selected image to create a binary mask that separated the target cells from the background. To ensure that the cropping was centred on the cells themselves, the centre of each cell was determined from the binary mask. Next, to determine the appropriate crop size for each image, the radius of each cell was measured using the SciPy packages, and we defined a bounding box through the centre and radius of each cell. Then the images were cropped according to their respective bounding boxes.

### Quantitative analysis of morphological features within cells

Quantitative cell morphological features (structural and biochemical features) were calculated from the 3D RI tomograms of individual cell images. Each cell was segmented from the surrounding medium based on a predetermined threshold RI value. We then calculated the cell volume (V) based on the number of voxels observed in each inner region of the cell. Next, we calculated the biochemical features’ protein density since we need the values of protein density and cell volume to calculate dry mass. The protein density was calculated by subtracting the medium’s RI from the divided cells’ RI. The difference between RI and protein concentration was divided by the increase in the RI values to calculate the protein density. After calculating the protein density, the dry mass of the cells was calculated. The calculated protein density (g/dL) was multiplied by the cell volume (V) and multiplied by 10^−2^ to obtain the dry mass in picogram (pg) units.

Centre coordinate points were measured using SciPy packages. Based on the centre coordinate points of each cell, we extracted cross-sectional images specific to that cell region by slicing the region centered on each cell. From the cross-sectional images, we measured the overall and nuclear component RI. The nuclear component RI focused on points with RI component values higher than 1.38. These quantitatively measured morphological features were then compared between each time point of sepsis recovery and the healthy cohorts.

### Spatial distribution of cellular shells

We extracted the cross-sectional images by slicing the region of each cell based on the centre coordinate points. The centre coordinates of each cross-sectional image were used to calculate the radius of the cell. Each cell was divided into eight elliptically shell regions. First, we calculated the Euclidean distance from the centre of the cell to each voxel and sorted all the voxels based on this distance. This created a ranked list from the closest to the furthest voxels. This list was then evenly divided into eight parts, each corresponding to a single shell. The region closest to the centre is called Shell 1, and the region furthest from the centre is referred to as Shell 8. This ensured a consistent distribution of voxels across each shell, effectively generating eight distinct ‘sub-regions’ within each cell. After generating the shells, the shell was converted into a binary mask. We applied a lower-intensity threshold to the original image to enhance the focus on cellular structures and reduce background noise. We then multiplied the binary mask of each shell to the original image to create a shell-specific image. This procedure effectively selected only the voxels within each specific shell, with all others set to zero for clear differentiation. The density of each shell was calculated by counting the number of non-zero voxels and dividing this by the total number of voxels in that shell^43^.

### Deep learning models for predicting the diagnosis and prognosis of sepsis

The models that predict the diagnosis and prognosis were developed based on the 3D images of cells: the diagnosis prediction model that differentiates between septic shock (T1) and healthy controls (H), and the prognostic prediction model that differentiates survival from non-survival at the septic shock time point (T1). To account for the variability of each cell, an input size of 210 × 276 × 276 pixels was individually centre-cropped according to the size of each cell. The images were then resized to the median size to maintain consistency in the input data for the model. Following the cropping step, the cropped images were min-max normalized to each image^44^. To align the input size of the deep learning model, all cropped images were resized to match the median size of the original image. Finally, the dataset was divided into training, validation, and test sets in an 8:1:1 ratio.

The model architecture was based on a modified DenseNet, which consists of an overall structure of 82 dense layers (Fig. S1a) divided into four dense blocks (Fig. S1b), with transition layers (Fig. S1c) placed between them for feature dimension reduction^45^. A cross-entropy loss and stochastic gradient descent algorithm with 16 mini-batch sizes were used to train the model. The cosine annealing method was used to determine the learning rate, which was set to an initial step size of 0.001 and a period of 64 epochs. Data augmentation and early stopping methods are applied to prevent model overfitting. Data augmentation, including random rotation processes and horizontal and vertical flips, was performed for each image once in every epoch. The early stopping method measured the performance with validation loss to terminate training, with a patience of 30.

To evaluate the performance of our model while accounting for the inherent cellular variability, we employed a bootstrapping method. This approach ensured the robustness of our models against patient-specific variances due to individual cellular differences. We ran 1000 iterations to estimate performance metrics AUROC to provide a reliable measure of the model’s predictive performance.

All deep learning processes were performed using PyTorch (version 1.13.0) on a server with two Intel Xeon Platinum 5253 processors, 128 Gb memory, and two NVIDIA Quadro RTX 8000 GPU 48 Gb, with CUDA version 10.0.

### Model validation with clinical features and the model itself

The relationships between morphological and clinical features, such as WBC count and neutrophil, lymphocyte, CRP, and cytokine levels, were evaluated. The mean RI of the cells was used to represent morphological features. Laboratory test results were extracted from EMRs. The following plasma cytokine levels were measured using the Human Magnetic Luminex® Discovery Kit (R&D Systems, Inc., Minneapolis, MN, USA)^46^ from the sampled blood: CXCL2/MCP-1, IL-10, IL-2, and TNF-alpha.

The gradient-weighted class activation mapping (Grad-CAM)^47^ algorithm was applied to derive the visual explanations by localising the image area that most influenced the decisions made using the deep learning model.

### Statistical analysis

Demographic and clinical variables and acquired cell counts were summarised for the entire cohort. Categorical variables are shown as frequencies and percentages, and continuous variables as medians with interquartile ranges.

Morphological features were compared using one-way analysis of variance (ANOVA) tests, and post-hoc analysis was performed using Student’s *t*-test for each time point of sepsis recovery and healthy controls. To compare the spatial distribution within cells, the density of each shell component was compared among the time points of sepsis recovery with those of healthy controls using ANOVA. The density of each shell component was also compared between cell images in the survival and non-survival groups taken at T1 using Student’s t-test. The Bonferroni correction was used to adjust for multiple comparisons.

The AUROC was used to measure the model performance. The AUROC was calculated by randomly selecting one to five cell images from each test set. The confidence interval (CI) of the AUROC was calculated using 1000 resamples. To compare conventional clinical indices, such as Systemic Inflammatory Response Syndrome (SIRS), Quick Sequential Organ Failure Assessment (qSOFA), Sequential Organ Failure Assessment (SOFA), and Modified Early Warning Score (MEWS), to predict the diagnosis or prognosis of sepsis, we depicted the previously known sensitivity and specificity values of each index on the same AUROC plots. Moreover, we plotted box plots to show the distribution of AUROCs with randomly selected cells to show the selection bias and variation of a random sampling of one to five cells.

Morphological and clinical features at each time point during sepsis recovery were plotted. We calculated Pearson’s correlation coefficients between morphological and clinical features to explore this association, including laboratory tests and cytokine levels.

Statistical analyses were conducted using the R software (version 4.2). Statistical significance is indicated as follows: *P < 0.05, **P < 0.01, and ***P < 0.001.

### Supplementary information

Supplementary information

## Data Availability

The raw dataset from our study can be accessed at https://drive.google.com/drive/folders/1qHjlh6OURBUALEfUr8cRIipZD94iebnW?usp=sharing.

## Code availability

The source code and software pipeline to reproduce our study can be accessed at https://github.com/DigitalHealthcareLab/22CellMorphologyLabelingTool.
