# Supplementary material for: Three-dimensional label-free morphology of CD8 + T cells as a sepsis biomarker
Source: Light Sci Appl. 2023 Nov 7;12:265. doi: 10.1038/s41377-023-01309-w (PMC10628166; doi:10.1038/s41377-023-01309-w)
Supplement: Supplementary file 1 — Supplementary information [file 41377_2023_1309_MOESM1_ESM.docx]

**Supplementary Information for**

**Three-dimensional label-free morphology of CD8+ T cells as a sepsis biomarker**

MinDong Sung^1+^, Jong Hyun Kim^2+^, Hyun-Seok Min^3+^, Sooyoung Jang^2^, JaeSeong Hong2, Bo Kyu Choi^2^, JuHye Shin^1^, Kyung Soo Chung^1∗^, Yu Rang Park^2∗^

^1^ Division of Pulmonary and Critical Care Medicine, Department of Internal Medicine, Yonsei University College of Medicine, Seoul, Republic of Korea

^2^ Department of Biomedical Systems Informatics, Yonsei University College of Medicine, Seoul, Republic of Korea

^3^ Tomocube, Inc, 155 Sinseong-ro, Shinsung-dong, Yuseong-gu, Daejeon, South Korea

^+^ MinDong Sung, Jong Hyun Kim, and Hyun-Seok Min contributed equally to this paper.

* Correspondence to: [chungks@yuhs.ac](mailto:chungks@yuhs.ac); yurangpark@yuhs.ac

**Supplementary Information Section 1. Inclusion and exclusion criteria for the sepsis cohort**

**Inclusion Criteria**

**Sepsis was defined as follows:**

(1) Patients with suspected infections,

(2) two or more criteria of the quick Sequential Organ Failure Assessment (qSOFA), which consists of respiratory rate ≥ 22 min^-1^, altered mentation, and systolic blood pressure ≤ 100 mmHg, were satisfied, and

(3) an acute change in total Sequential Organ Failure Assessment (SOFA) score ≥ 2 points due to the infection.

**Sepsis was defined as follows:**

(1) Patients who were diagnosed with sepsis, and

(2) whose vasopressor requires a mean arterial pressure of 65 mmHg or greater and a serum lactate level greater than 2 mmol L^-1^ in the absence of hypovolemia.

**Exclusion Criteria**

**Patients with the following conditions were excluded:**

(1) age < 19,

(2) pregnant or lactating,

(3) active cancer status,

(4) acute stroke,

(5) acute cardiovascular disease,

(6) acute burns,

(7) acute gastrointestinal bleeding or bleeding within the last three months,

(8) taking immunosuppressive drugs after organ transplantation,

(9) taking immunosuppressive drugs for autoimmune disease,

(10) previously diagnosed immunodeficiency conditions, or CD4 cell counts below 350 G L^-1^,

(11) neutropenia (neutrophils <500 G L^-1^) or if the neutrophils were 500–1000 G L^-1^ due to chemotherapy and were expected to decrease,

(12) diagnosed with adrenal dysfunction,

(13) prescribed a steroid equivalent to or greater than 0.5 mg kg^-1^ day^-1^ prednisone,

(14) active tuberculosis,

(15) cystic fibrosis,

(16) post-traumatic,

(17) who needed immediate surgery, and

(18) the state of Do-Not-Resuscitate (DNR)

**Supplementary Information Section 2. Deep learning model architecture**

**
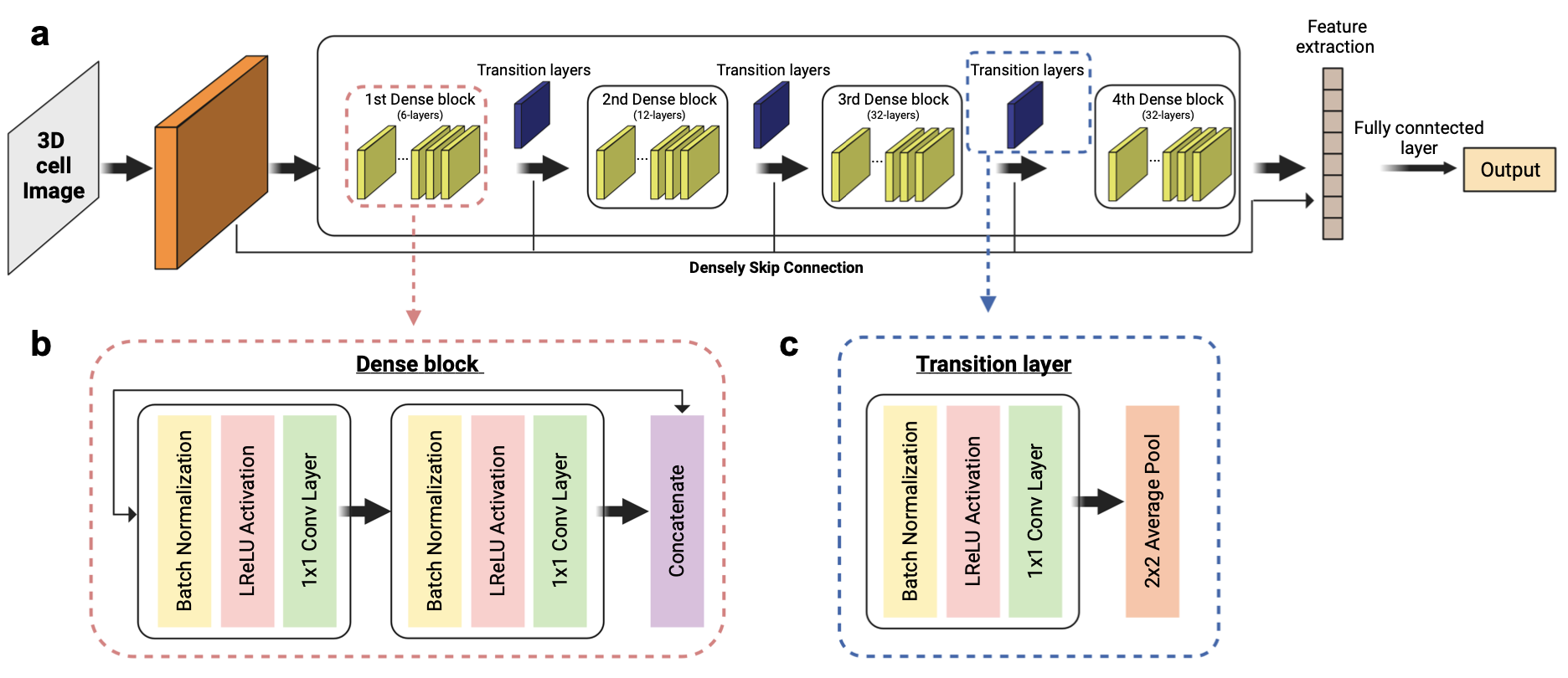
**

**Fig. S1. Three-dimensional deep learning model architecture for 3D cell image classification. a.** The overall network structure, including 82 dense layers segmented into four dense blocks connected by three transition layers. Each dense block contains 6, 12, 32, and 32 dense layers, respectively. **b.** Detailed view of a single dense block, detailing the two-step process: the bottleneck layer and the feature map generation layer. The bottleneck layer includes batch normalisation, Leaky Rectified Linear Unit (LReLU) activation, and a 1 × 1 convolutional layer to reduce the input features. The feature map generation layers use batch normalisation, LReLU activation, and a 3 × 3 convolutional layer to generate a new feature map. **c.** The transition layers, positioned between dense blocks, reduce the dimensionality of acquired features. This layer includes batch normalisation, LReLU activation, a 1 × 1 convolutional layer, and a 2 × 2 average pooling layer.

We implemented a deep learning model to perform classification tasks on 3D cell image data using a modified architecture of the 3D Dense Convolution Network (DenseNet). The architecture of the 3D DenseNet consists of 82 dense layers, four dense blocks, and connected by three transitional layers. The dense blocks are composed of 6, 12, 32, and 32 dense layers, respectively. Each dense layer inherits the feature maps of all previous layers, allowing new features to be extracted without losing information.

Two steps are performed within each dense block. In the first step, the input features are reduced through a bottleneck layer, which includes batch normalisation, Leaky Rectified Linear Unit (LReLU) activation, and a 1 × 1 convolutional layer. The second step generates a new feature map through batch normalisation, LReLU activation, and a 3 × 3 convolutional layer. Processed feature maps are then concatenated with feature maps from previous layers, allowing the network to learn new features while maintaining all previous features. Transition layers are located between the dense blocks, which conduct the dimensional reduction of acquired features. Transition layers comprise batch normalisation, LReLU activation, a 1 × 1 convolutional layer, and a 2 × 2 average pooling layer. The 1 × 1 convolutional layer decreases the number of features, and the 2 × 2 average pooling layer reduces the size of the feature map by half. Finally, feature extraction is performed in the last dense block. The extracted features are then passed through a fully connected layer to make predictions for each class.
